# Evaluation of Direct Medical Costs Associated With High-Risk Febrile Neutropenia Among Cancer Patients in a Tertiary Care Center

**DOI:** 10.7759/cureus.102614

**Published:** 2026-01-30

**Authors:** Sairam BVSN, Mirunalini Ravichandran, Smita Kayal, Kiruthika S

**Affiliations:** 1 Pharmacology, Jawaharlal Institute of Postgraduate Medical Education and Research, Puducherry, IND; 2 Medical Oncology, Regional Cancer Centre, Jawaharlal Institute of Postgraduate Medical Education and Research, Puducherry, IND; 3 Pharmacology, Jawaharlal Institute of Postgraduate Medical Education and Research, Karaikal, IND

**Keywords:** adverse effect, chemotherapy, cost-analysis, fever with neutropenia, pharmacoeconomics

## Abstract

Introduction

The onset of febrile neutropenia (FN) demands dose reduction and usually temporary halting of chemotherapy, which could then affect the outcome of cancer treatment. In this study, we wanted to quantify the drugs used in treating febrile neutropenic episodes, the costs incurred as a result, and the outcomes of these episodes.

Methods

The study was a prospective observational study. Patients of either sex, of any age, and diagnosed with high-risk FN following cancer chemotherapy and hospitalized during the period of study were included. A total of 46 patients with 50 episodes of FN were enrolled. Data were collected from the department database and daily clinical notes and were entered into a pre-designed proforma. The drugs prescribed were classified as antibiotics and supportive medications. The drug cost of each episode was then calculated. The data were analysed using descriptive statistics.

Results

The mean age of participants was 26.66 years, with a median length of hospital stay of 10 days. This provides an estimate of the drug-related costs. A total of Rs. 8,21,731 was spent on drugs for the management of 50 episodes, and the average cost of an episode was Rs. 16,434. Of the total cost, Rs. 7,00,300 (85.22%) was spent on antimicrobials.

Conclusion

Considering the complexities of medical decision-making and quality of care, the role of cost needs to play a major role in therapeutic options. There is a need to develop value-based policies to achieve the best clinical outcomes while accounting for cost-effectiveness and polypharmacy.

## Introduction

Febrile neutropenia (FN) is a commonly encountered life-threatening complication of cancer chemotherapy. It is defined as an oral temperature of >38.3˚C (101˚F), with an absolute neutrophil count (ANC) less than (or expected to fall below) 500/mm³ [1]. It is the dose-limiting toxicity in the therapy of many cancers, most notably haematologic malignancies such as leukaemias [2]. The onset of FN thus demands dose reduction and usually temporary halting of chemotherapy, which could affect the outcome of cancer treatment [3].

FN is a medical emergency that requires high clinical suspicion, prompt diagnosis, and immediate treatment. Moreover, with a mortality of about 3-5% in children [4-7] and over 10% [8-11] in adults, FN is a significant cause of death in cancer patients [12]. This risk worsens in the presence of additional comorbid conditions. Another hurdle in the management of FN is the identification of etiology. The causative organism may be isolated in only 30% of cases or fewer [8,13-15]. In cases where microbiological identification was possible, the most common organisms are gram-positive bacteria (Streptococci viridans group, *Streptococcus pneumoniae*, Group A Streptococci such as *Streptococcus pyogenes*, Staphylococci - coagulase-negative, *Staphylococcus aureus* including methicillin-resistant *Staphylococcus aureus* (MRSA), and Enterococcus), followed by gram-negative bacteria (*Escherichia coli*, *Klebsiella pneumoniae*, *Pseudomonas aerugiunosa*) [16]. Candida and Aspergillus are the usual fungal agents [17]. Such cases are classified as “microbiologically documented infections” (MDIs).

The focus of infection is another factor influencing the course of FN. Like MDIs, cases are classified as “clinically documented infections” (CDIs) if a focus of infection is clinically or radiologically determined. Once again, not all patients may have an identifiable infectious focus; it is identified in around 40% of cases [18,19], and fever is often the only sign or symptom of infection [20]. As FN is an emergency, and the etiologies are often uncertain, treatment of FN is initiated empirically and may later be modified to narrow-spectrum antimicrobials if the pathogen and its susceptibility are determined. However, since isolation is not possible in many cases, treatment is continued to provide broad-spectrum coverage until the febrile episode resolves. Guidelines have been formulated at both the institute and international levels to provide adequate and appropriate antimicrobial coverage during empirical treatment of FN, and to level up antibiotics in cases of suspected resistance [21].

Apart from the burden it places on the healthcare system, owing to its morbidity, mortality, and difficulty in treatment, FN also incurs significant expenses and is regarded as an expensive side-effect of cancer chemotherapy. Neutropenia-related costs account for up to 8% of total cancer costs in adult patients and 27% of costs in paediatric patients [22], and severe episodes requiring hospital admission further impact these patients financially.

To counter the obstacles in managing FN, various risk-stratification systems have been formulated, such as the Multinational Association of Supportive Care in Cancer criteria (MASCC) [1] and the Clinical Index of Stable Febrile Neutropenia score (CISNE) [23]. Stratifying patients into low- and high-risk categories optimises treatment appropriate to their clinical status and risk for complications - patients with low risk can be managed with oral antibiotics on an outpatient basis. In contrast, high-risk patients require hospitalisation in addition to parenteral therapy [1,16]. This thus prevents unnecessary hospitalisation, saving hospital resources and reducing patient expenditure [24]. Along with antimicrobials, supportive medications such as antipyretics (paracetamol), drugs for symptom relief (anti-diarrheal agents, topical anaesthetic medication), and granulocyte-colony stimulating factor (G-CSF) are administered in select cases to improve blood cell counts.

There have been studies recognising the economic burden of FN and have analysed the total cost of hospitalisation, as well as of the drug therapy alone, both around the world and in India [22,25-28]. In our study, we aimed to quantify the drugs used in the treatment of febrile neutropenic episodes at our centre, a tertiary care centre in South India, and the associated costs, as well as the outcomes of these episodes.

The article was made available in Research Square, and it was subsequently withdrawn.

## Materials and methods

The study was designed as a prospective observational study. The study was conducted in the Department of Medical Oncology at a tertiary care centre in South India, from July 2023 to September 2023. The study was conducted in accordance with the principles of the Declaration of Helsinki and was approved by the Institute Ethics Committee (JIP/IEC-OS/2022/235, dated 11.08.2022). Written informed consent was obtained from all patients enrolled in the study.

The study population was patients of either sex, of any age, diagnosed with cancer and classified as high-risk FN (according to MASCC criteria and clinical judgement), and hospitalised during the period of study, and patients with Eastern Cooperative Oncology Group (ECOG) 3 or more were excluded. As this was a short-term studentship project, the study duration was three months. Hence, all patients fulfilling the eligibility criteria were included during the study period. FN was defined as a condition in which patients had an oral temperature of >38.3˚C (101˚F), with an ANC less than (or expected to fall below) 500/mm³ [1]. There was no exclusion criterion. All patients fulfilling the study criteria were admitted to the ward.

According to the Department of Medical Oncology's past records, approximately 250 to 300 episodes of FN were managed annually in the wards. Based on the eligibility criteria, we expected to enroll about 50-60 episodes of FN during our study duration of three months. Hence, the sample size was calculated based on the hospital statistics for feasibility and was not calculated using a formula or software. However, considering an incidence of 10% FN cases (based on hospital statistics) with 5% absolute precision and 80% power, the sample size was calculated to be 60, using OpenEPI v3.01 (Andrew G. Dean, Kevin M. Sullivan, Minn M. Soe, Atlanta, GA, USA), assuming no attrition during the study period. Considering a study period of three months, we were able to include only 50 episodes of FN. The last patient recruited was followed up for a minimum of two weeks from the onset of FN. The sampling method adopted for this study was a consecutive sampling technique.

Episodes of FN were managed according to department protocol, which included baseline blood cultures and blood counts, followed by the institution of empirical broad-spectrum antibiotics (intravenous/oral) with or without G-CSF supplementation. Patients were monitored daily for clinical status and lab parameters as indicated, till resolution of fever and recovery of counts. Treatment was initially started empirically, with cefepime 2 g intravenous (IV), eight hourly, acyclovir 400 mg oral, twice daily, and voriconazole 200 mg oral twice daily, with escalation to second-line antibiotics such as meropenem 1 g IV, eight hourly, vancomycin 1 g IV, twice daily, or linezolid 600 mg IV, twice daily. The therapy was modified based on the culture and sensitivity reports. Further escalation to restricted antibiotics, such as colistin 3 million IU IV, eight hourly, and tigecycline 50 mg IV, twice daily, was done in episodes that did not respond to second-line drugs. Treatment was started with voriconazole 200 mg oral, twice daily as empirical therapy. Escalation to posaconazole 300 mg orally, once daily, conventional amphotericin B at 1 mg/kg/day IV, or caspofungin (50 mg IV, daily) was done in cases of unresponsiveness to first-line therapy.

Data was prospectively collected from the department database and daily clinical notes and was entered into a pre-designed proforma. The details collected included demographic details, diagnosis, treatment history, and daily prescription lists. The drugs prescribed were classified as antibiotics and supportive medications. The data collected through the proforma was then organised in Microsoft Excel^®^ (Microsoft, Redmond, WA, USA). Information regarding the cost of each drug was obtained from the institute’s dispensing pharmacy. The drug cost of each episode was then calculated.

The parameters studied included demographic characteristics such as age, sex, date of admission, ANC at presentation, ECOG performance status, nutritional status; disease characteristics such as cancer diagnosis, chemotherapy protocol, day of onset of FN, prophylactic use of G-CSF, type of FN; treatment characteristics such as length of hospital stay, drugs administered (from Daily Medications List) and drug costs; and clinical outcome.

Baseline characteristics of the cases were analysed using descriptive statistics. Categorical data are described using percentages and frequencies, and are compared using the chi-square test or Fisher’s exact test. Continuous data is expressed as mean with SD or median with range, and was analysed by Student's t-test or the Wilcoxon test, as applicable. Analysis was carried out at 5% level of significance, and a p-value <0.05 was considered statistically significant. All calculations were performed using STATA v14.0 (StataCorp., College Station, TX, USA).

## Results

A total of 63 episodes of FN were identified as potentially eligible for our study from September 2022 to September 2023. All 63 episodes were examined for eligibility, and 50 episodes were confirmed as eligible. All 50 eligible episodes were included in the study, and all were followed up on for the duration of the study. Thus, we had 46 individual patients with a total of 50 episodes of FN. All recruited patients had an ANC of less than 500/mm³ or were expected to fall below 500/mm³ and a temperature greater than 38.3˚C (101˚F) at the time of admission, with a mean ANC at presentation of 250.20 (SD 204.84) per mm³.

The demographic characteristics of the enrolled patients are shown in Table 1. The mean age of all patients was 26.66 (SD 13.28) years, while the mean age of 11 (22%) paediatric patients was 10.91 (SD 4.74) years, and that of adult patients was 33.67 (SD 10.31) years. The study group comprised 22 (48%) male patients and 24 (52%) female patients. Forty-two patients (91.3%) were diagnosed with haematologic malignancies, and four patients (8.7%) with solid tumours. Twenty-four episodes (52.2%) required ICU admission during hospital stay. The overall length of hospital stay for all patients showed a median of 10 days with an IOR of 7-15 days. The median day of onset of FN was nine days (IQR, 7-12). The day of FN onset here refers to the interval between the first dose of chemotherapy in the cycle and the first occurrence of FN.

**Table 1:** Demographic characteristics of the study participants Values are represented as n (%). *Represent the total number of admissions based on the number of episodes of febrile neutropenia.

Clinical Characteristics	N=46, n (%)
Age (years)	
Less than 18	11 (24)
19 and above	35 (76)
Gender	
Male	22 (47.8)
Female	24 (52.2)
Cancer diagnosis	
Haematologic	42 (91.3)
Non-hematologic	4 (8.7)
Admission type (N=50)^*^	
General inpatient ward	26 (52)
Intensive care unit	24 (48)

The various chemotherapy regimens received by the participants during the FN episodes are shown in Table 2, which includes chemotherapy regimens for leukaemias, lymphomas, and other cancer types. 

**Table 2:** Chemotherapy regimens received during febrile neutropenia episodes *Others included R-CHOP regimen - rituximab, cyclophosphamide, hydroxydaunorubicin, vincristine, prednisolone, ATRA regimen - all-trans retinoic acid, Packer regimen, EMACO regimen - etoposide, methotrexate, actinomycin D, cyclophosphamide, vincristine, ICE regimen - ifosfamide, carboplatin, etoposide, R-COPADM regimen - rituximab, cyclophosphamide, vincristine, prednisolone, adriamycin, methotrexate.

Chemotherapy Regimen	N=50, n (%)
Daunorubicin/Cytarabine induction regimen	20 (42)
High-dose cytarabine (HiDAC)	9 (18)
Berlin-Frankfurt-Munster (BFM) 95 regimen	6 (12)
Others*	15 (36)

The types of infections encountered in the FN episodes have been described in Table 3. CDIs are those infections in which an identifiable focus of infection is present. Microbiologically documented infections are those infections in which the etiological organism has been isolated from a suitable sample (blood, urine, stool, wound, etc.). Infections are classified as fever of unknown origin (FUO) if there is neither an identifiable infectious focus nor any detectable organism. The most common type of infection was CDIs, accounting for 19 (38%) out of 50 episodes.

**Table 3:** Type of infection in febrile neutropenia episodes *Total N=50 episodes for 46 participants in this study.

Type of Infection	N=50*, n (%)
Clinically documented infections (CDIs)	19 (38)
Microbiologically documented infections (MDIs)	3 (6)
Both CDI and MDI	11 (22)
Fever of unknown origin (FUO)	17 (34)

The identified foci of infection are described in Table 4. There were 30 episodes (60%) that had an identifiable focus of infection. The most common focus was the gastrointestinal tract, 22 episodes (44%). The various organisms isolated in cultures from the FN episodes are shown in Table 5. Approximately 14 episodes (28%) showed positive cultures, with gram-negative bacilli (12 out of 14 episodes) being the most frequently isolated.

**Table 4:** Focus of infection identified in febrile neutropenia episodes CDI, clinically documented infections; CMDI, clinically and microbiologically documented infections.

Parameter	N=50, n (%)
Focus of infection (CDIs and CMDIs)	
Gastrointestinal	22 (44)
Groin ulcer	3 (6)
Pulmonary	5 (10)
No focus identified	20 (40)

**Table 5:** Organisms isolated from culture in febrile neutropenia episodes MDI, microbiologically documented infections; CMDI, clinically and microbiologically documented infections.

Parameter	N=50, n (%)
Organism isolated (MDIs and CMDIs)	14 (28)
*Klebsiella pneumoniae*	5 (10)
*Escherichia coli*	4 (8)
*Bacillus cereus*	1 (2)
*Brevundimonas diminuta*	1 (2)
*Candida*	1 (2)
*Pseudomonas aeruginosa*	1 (2)
*Acinetobacter baumannii*	1 (2)
No identified organism	36 (72)

The cost of the medications prescribed to patients was determined, and the costs of antimicrobial drugs, supportive medications, and total drugs were calculated per episode of FN, as well as the total cost for 50 episodes, as described in Table 6. The mean drug cost per episode was Rs. 16,434.60/episode, and the mean drug cost per day of hospital stay was Rs. 1,475.28/day. A total of Rs. 8,21,731 was spent on drugs for the management of 50 episodes of FN. Of the total cost, Rs. 7,00,300 (85.22%) was spent on antimicrobials. Meropenem, the most prescribed antimicrobial in our study, alone accounted for Rs. 2,10,855, which was 30.11% of antimicrobial costs and 25.66% of total drug costs.

**Table 6:** Cost of treatment of febrile neutropenia ₹, Indian Rupee symbol; FN, febrile neutropenia.

Parameter	Value in ₹
Cost per episode	
Mean drug cost	16434.60
Mean cost of antimicrobial use	14006.00
Mean cost of supportive medication	2428.61
Cost of treatment of 50 episodes of FN	
Total cost of drugs	8,21,731
Total cost of antimicrobials	7,00,300
Total cost of supportive medications	1,21,431

A cost of Rs. 1,21,431 was incurred for supportive medications during these episodes. Notably, human albumin was only used in two cases but accounted for Rs. 38,500 (31.71%) of these costs. Paracetamol (IV - Rs. 12,836.13 and oral - Rs. 137.10) accounted for 15.2% of the supportive cost. G-CSF was used in 16 episodes, incurring a cost of Rs. 6,900.55, which accounted for 8.1% of the supportive care cost.

We found that the antimicrobials with highest expenditure per day of use were liposomal amphotericin B (for 150 mcg IV once daily, Rs. 4050/day), caspofungin (for 50 mg IV once daily, Rs. 1320/day), meropenem (for 1 g IV thrice daily, Rs. 584.64/day), colistin (for 3 million IU IV thrice daily, Rs. 398.16/day), and tigecycline (50 mg twice daily, Rs. 322.56/day). Amongst the supportive medications, human albumin (for 100 mL, 20% w/v, IV once daily, Rs. 3500/day), paracetamol (for 1 g IV thrice daily, Rs. 86.79/day), and G-CSF (for 300 mcg SC once daily, Rs. 85.88/day) had higher costs per day.

## Discussion

Forty-six participants and 50 episodes of FN, defined as the presence of fever >101˚F and an ANC below 500/mm^3^, were consecutively recruited into this study. Patient characteristics, disease characteristics, treatment characteristics, and clinical outcome were assessed by accessing patient records and the daily medications list. The mean age of the participants was 26.66 (SD 13.28) years, with a median length of hospital stay of 10 days (range, 7-15 days). Acute myeloid leukaemia (AML) was the cancer diagnosis, with overall haematologic malignancies accounting for almost 90% of episodes. Foci of infection were found in 60% of cases, with gastroenteritis (44%) being the most common focus. In the study by Cortés et al. [19], an infectious focus was identified in 65% of patients, and it was found that the urinary tract (19%) and gastrointestinal tract (19%) were the most common foci of infection.

The study by Mandal et al. [13] found that isolation of organisms was possible in 29.1% of cases, and gram-negative bacilli were found in 61.5% of cultures. Similarly, in our study, isolation was possible in 28% of cases, and gram-negative bacilli were isolated in 85.7% of positive cultures (12 out of 14 MDIs).

Roy et al. [28] calculated the average total cost of therapy of an FN episode to be Rs. 4,700. However, their study included both low-risk and high-risk patients, and treatment was on an outpatient basis. Our study found the average cost of treatment of high-risk FN to be Rs. 16,434. Moreover, in the study by Roy et al., G-CSF accounted for the majority (76-98%) of the costs, whereas in our study, antimicrobials accounted for more than 85% of the costs, and G-CSF accounted for only 1.5% of the total cost. This may be owed to the fact that G-CSF was significantly more expensive at the time of their study.

We were unable to achieve the sample size during the available study period, and hence, post-power analysis was not performed. The limitations of our study were that it did not account for hospitalisation costs, room charges, investigation costs, consultation charges, etc. It also does not include other indirect costs of treatment, such as loss of wages and transportation. Other limitations include a small sample size and a short study duration. The costs of drugs, depending on whether they are government-aided or not, also vary from centre to centre. 

## Conclusions

This study describes the drugs used in the treatment of febrile neutropenic episodes and estimates the drug-related costs of managing FN. From the total cost spent on managing febrile neutropenic episodes, approximately 85% was allocated to antibiotics. Meropenem was the most prescribed antimicrobial in our study. Antimicrobials with the highest expenditure per day of use were liposomal amphotericin B. Among the supportive medications, G-CSF was used in 32% of the episodes and accounted for 8.1% of the supportive care cost. Considering the complexities in medical decision-making and quality of care, the role of cost needs to take a major role in therapeutic options, leading to value-based policies. Guidelines could be framed to rationalise antimicrobial use and develop cost-effective drug therapies that decrease the economic burden on both patients and the healthcare system.
